# A Dizzying, Complex Spectacle

**DOI:** 10.3201/eid2804.AC2804

**Published:** 2022-04

**Authors:** Byron Breedlove

**Affiliations:** Centers for Disease Control and Prevention, Atlanta, Georgia, USA

**Keywords:** art science connection, emerging infectious diseases, art and medicine, about the cover, a dizzying, complex spectacle, John August Swanson, The Carousel, public health, animals, pathogens, viruses, bacteria, fungi, parasites, zoonoses, epidemiology, sylvatic cycle, synanthropic cycle, carousel, serigraph

**Figure:**
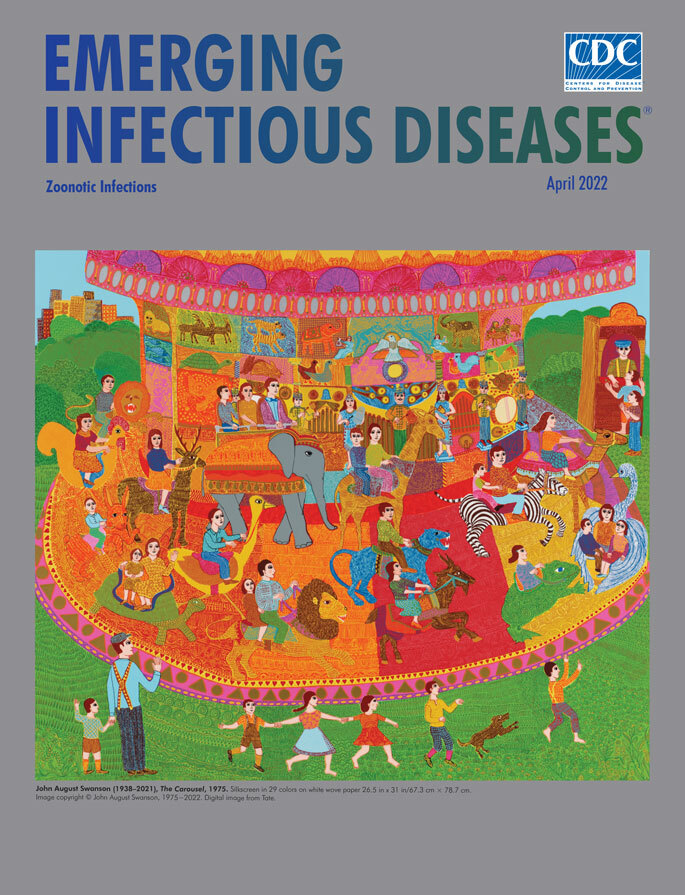
**John August Swanson (1938–2021) *The Carousel*, 1975.** Silkscreen in 29 colors on white wove paper 26.5 in x 31 in/67.3 cm × 78.7 cm. Image copyright © John August Swanson, 1975‒2022. Digital image from Tate.

When American artist John August Swanson died in September 2021, he left an extensive body of artwork focusing on social justice issues, religious themes, and everyday activities. The Theo Arts Gallery, Boston University School of Theology, notes, “His art reflects the strong heritage of storytelling he inherited from his Mexican mother and Swedish father. John August Swanson’s narrative is direct and easily understood. He addresses human values, cultural roots, and a quest for self-discovery through visual images.” His artwork is housed in collections at the Smithsonian’s National Museum of American History, National American Art Museum, and National Air and Space Museum; the Art Institute of Chicago; Tate Gallery (UK); the Vatican Museums’ Collection of Modern Religious Art; and Emory University’s Candler School of Theology.

Swanson painted with oils, watercolors, and acrylics; created lithographs and etchings; and made colorful, detailed serigraphs—an artists’ term for silkscreen painting. According to Emory University’s Pitts Theology Library, “These serigraphs necessitate an advanced level of technical acumen and typically feature 30 to 60 separate colors, each of which requires a separate stencil drawn by the artist. Swanson’s elaborate serigraph process results in pieces that have unique textures and colors that are characteristic of his mastery of this medium.” 

Swanson’s *The Carousel*, this month’s cover image, is a vibrant serigraph depicting the popular amusement ride—sometimes also called a merry-go-round—and illustrating a certain association and interaction between humans and animals. It is a dizzying, complex spectacle imbued with 29 distinct colors and intricate patterns that may be missed without scrutiny. Many animals, including the elephant, camel, lion, and zebra, are rendered in colors similar to their natural tones, but Swanson’s blue tiger near the center breaks that convention. Detailed textures and whirling shapes enliven fur, scales, shells, and feathers, rich brocades that contrast with the smooth-skinned elephant and white and black zebra. 

Swanson decorates the inner wall of the carousel—which hides its engine—with more images of animals and iconography of religion. The floor is partitioned by distinct brightly colored wedges and repeating shapes that continue around the platform. More repeating patterns decorate the carousel’s upper and lower edges. 

The people depicted in the serigraph, save for their age differences, are largely uniform. Riders and spectators share skin tone, facial feature shape, dark eyes, and hair color. Their clothing, featuring more patterns, swirls, and colors, distinguishes one from another. Except for the trio at the ticket booth, most people and animals are facing counterclockwise, the direction in which carousels typically rotate in North America. The background whorls of unnaturally verdant grass merging into a panoply of trees, capped by glimpses of pale blue sky, and a small cluster of buildings in the upper left all suggest that the location is a city park. 

Swanson’s insouciant image, including domesticated and wild animals from around the world, also works as a metaphor for humankind’s relationships with and connections to other animals through the lens of zoonotic disease. An observer watching such a carousel would see the figures emerge and reemerge, not unlike disease detectives tracking zoonotic diseases. Zoonoses, an unavoidable consequence of the interactions among humans and animals, are caused by harmful germs such as viruses, bacteria, parasites, and fungi. The Centers for Disease Control and Prevention notes that more than 6 out of every 10 known infectious diseases in people come from animals and that 3 out of every 4 new or emerging infectious diseases in people come from animals. Knowing which animals could have zoonotic diseases proves challenging because both domesticated animals and wildlife may appear well, act healthy, yet carry lethal pathogens.

Like Swanson’s cycling carousel, emerging and reemerging zoonotic diseases―which are transmitted from animals to humans―and the pathogens that cause them, also occur in cycles as they propagate among different ecosystems. The sylvatic cycle occurs in natural settings among wild animals and vectors; the synanthropic cycle occurs where domestic and companion animals live in close association with humans. Other cycles of transmission are human-to-human transmission and human-to-animal transmission. Many interrelated factors, including climate change, modern agricultural practices, destruction of natural habitats, urbanization, and seasonality of infectious diseases, contribute to the complex and dizzying array of zoonoses that pose a challenge to public health.
